# Contextual memory engrams, and the neuromodulatory influence of the locus coeruleus

**DOI:** 10.3389/fnmol.2024.1342622

**Published:** 2024-02-05

**Authors:** Stephanie L. Grella, Tia N. Donaldson

**Affiliations:** ^1^MNEME Lab, Department of Psychology, Program in Neuroscience, Loyola University Chicago, Chicago, IL, United States; ^2^Systems Neuroscience and Behavior Lab, Department of Psychology, The University of New Mexico, Albuquerque, NM, United States

**Keywords:** locus coeruleus, memory-updating, hippocampus, contextual memory, neuromodulation, engram, norepinephrine, remapping

## Abstract

Here, we review the basis of contextual memory at a conceptual and cellular level. We begin with an overview of the philosophical foundations of traversing space, followed by theories covering the material bases of contextual representations in the hippocampus (engrams), exploring functional characteristics of the cells and subfields within. Next, we explore various methodological approaches for investigating contextual memory engrams, emphasizing plasticity mechanisms. This leads us to discuss the role of neuromodulatory inputs in governing these dynamic changes. We then outline a recent hypothesis involving noradrenergic and dopaminergic projections from the locus coeruleus (LC) to different subregions of the hippocampus, in sculpting contextual representations, giving a brief description of the neuroanatomical and physiological properties of the LC. Finally, we examine how activity in the LC influences contextual memory processes through synaptic plasticity mechanisms to alter hippocampal engrams. Overall, we find that phasic activation of the LC plays an important role in promoting new learning and altering mnemonic processes at the behavioral and cellular level through the neuromodulatory influence of NE/DA in the hippocampus. These findings may provide insight into mechanisms of hippocampal remapping and memory updating, memory processes that are potentially dysregulated in certain psychiatric and neurodegenerative disorders.

## 1 Philosophical perspectives and contextual representations

### 1.1 Negotiating space

For centuries, we’ve sought to understand how the brain processes higher cognition, particularly in extracting meaning from a seemingly chaotic environment. In psychology, Gestalt theory, derived primarily from observations regarding the interaction between an organism and its environment, views perception from a perspective where we create holistic representations of the environment, and holds that these unified representations are distinct from the sensory inputs from which they are created. The brain’s specific method for unifying these inputs remains unclear, such as in spatial cognition where we determine features and angles to mentally represent the space around us to form a coherent layout that we are a part of and integrate this with movement. We often take for granted the neural sophistication necessary simply to avoid collisions during navigation. And yet as sophisticated as animal movement is, we sometimes still bump into things. How do these errors arise? The negotiation of space, in other words, movement, is often goal-directed, highlighting the importance of adaptive place learning, linking spatial learning and memory to animal movement (Collins et al., 2006; Mueller and Fagan, 2008; Nathan et al., 2008; Paul et al., 2009; Fagan et al., 2013; Gautestad et al., 2013).

Borrowed from mathematics, the term isomorphism is used to describe relatedness or formal correspondence between systems or entities (Gallistel, 1989). Gallistel (1990) described aspects of the environment (e.g., an object in your path), and the neural processes that function to direct an organism’s behavior in such a way that allows the individual to cope with the environment in an adaptive way (e.g., maintaining or changing trajectory to avoid the object), as functioning isomorphisms. He made the important assertion that creating representations alone is not interesting, what is interesting is how the brain operates in a manner of combinatorial processing to give rise to these spatial representations while also integrating motivational and attentional states, internal and external sensory information, along with movement, to promote adaptive behavior (Gallistel, 1989; Mizumori et al., 2000). Being able to remember significant locations is evolutionarily imperative to survival. Animals including humans use these skills to complete tasks such as finding food, finding a mate, and seeking shelter. And in the modern world, it has been shown that people with higher lifetime global position system (GPS) experience have worse spatial memory when they have to navigate without a GPS (Dahmani and Bohbot, 2020).

The ability to navigate is contingent on neural systems that are responsible for encoding moment to moment changes in an animal’s place, direction, or heading within space (O’Keefe and Dostrovsky, 1971; O’Keefe and Nadel, 1978). Navigational strategies have been demonstrated to be highly organized and conserved across species (O’Keefe and Nadel, 1978). The specific navigational strategy employed is dependent upon the sources of information available to the animal. These sources of information are categorized as *environmental*, or *self-motion* cues (Gallistel, 1990). Cues from the environment (e.g., visual, auditory, olfactory) are often stable, salient, and can be reliably used to maintain orientation within space, which is referred to as *piloting* (Gallistel, 1990; Maaswinkel et al., 1999). In contrast, animals can navigate via *path integration*, also called *dead reckoning*, which involves summing vectors of distance and direction as one travels (McNaughton et al., 2006), using internally generated self-motion (idiothetic) cues (e.g., vestibular input, optical flow) (Maaswinkel et al., 1999). When navigating, especially within a novel environment, it is most efficient to use a combination of environmental and idiothetic cues.

Based on early work with laboratory rats in mazes, it became clear that rats could complete certain spatial tasks, not by remembering a series of turns or responses, but by somehow orienting themselves with respect to landmarks and cues. Edward Tolman coined the term *cognitive map* in Tolman (1948) to describe a mental representation of the environment, akin to a paper map. This map guides navigation based on Euclidean metrics and included flexible navigation from unfamiliar locations, seen as a *relational representation*. The discovery of spatially responsive cells in the hippocampus (O’Keefe and Dostrovsky, 1971) supported this theory and demonstrated that this structure played a crucial role in spatial and contextual processing. Damage to the hippocampus impairs such flexible navigation, aligning with the idea that the hippocampus is the neural basis for this relational representation, and is involved in creating global representations of the environment.

How does the brain represent space and is spatial memory innate or shaped by experience? Questions such as these gave rise to the *geometric module* (Cheng and Newcombe, 2005) and *cognitive map* theory (Tolman, 1948), notions originating from the ideology that our brains are preconfigured, or at least semi-preconfigured, with navigational hardware (Ainge and Langston, 2012). The idea of an *a priori* representation of space dates back to Kant (1922), Burnham (2008), and Janiak (2009), who opposed Hume (1738) empirical view. Kant proposed that space is not empirically testable, and surmised that since we cannot perceive the *absence of space* we must, therefore, have innate *knowledge of space* (Burnham, 2008). Whether or not we possess the faculties to process our surroundings innately as the nativist Kantian perspective would suggest, or whether it is a result of acquired experience as empiricist Jean (1967) would argue, we do seem to be able to form coherent neural representations of the space we traverse. In fact, there is even evidence to suggest that we can form representations of the space we have not yet traversed, but plan to Maurer and McNaughton (2007), Dragoi and Tonegawa (2011, 2013), Azizi et al. (2013), Cona and Ursino (2015), and Ólafsdóttir et al. (2015). Some have referred to these representations as our *contextual code* (Nadel and Willner, 1985; Teyler and DiScenna, 1986; McNaughton et al., 1996). Understanding this code, and what is specifically being encoded within these contextual representations (e.g., space and time) has been the goal of many scientists in the last century. And while significant progress has been made, there remains much to decipher.

## 2 Hippocampal contextual code

### 2.1 Memory traces

The last 50 years have provided us with compelling evidence that the hippocampus is essential in processing spatial and contextual information (Hirsh, 1974; O’Keefe, 1976; Kim and Fanselow, 1992; Phillips and LeDoux, 1992; Holland and Bouton, 1999; Fanselow, 2000; Burgess et al., 2001, 2002; Guzowski and Worley, 2001; Schmolck et al., 2002; Anderson et al., 2003; Rudy et al., 2004; Vazdarjanova and Guzowski, 2004; Smith and Mizumori, 2006; Acheson et al., 2012; Maren et al., 2013; Nees and Pohlack, 2014; Sadeh et al., 2014; Smith and Bulkin, 2014). The hippocampus is divided into subregions that include the cornu ammonis (CA1, CA2, and CA3) and the dentate gyrus (DG). Lesions to the hippocampus produce severe spatial memory impairments in rats (Morris et al., 1982; Sutherland et al., 1982; Kesner et al., 1989), birds (Colombo et al., 1997; Fremouw et al., 1997), non-human primates (Murray et al., 1998), and humans (Bohbot et al., 1998). In rats, hippocampal lesions have been shown to disrupt spatial performance in a variety of behavioral tasks (Olton et al., 1979; Morris et al., 1982; Rawlins and Olton, 1982; Jarrard, 1993; Pearce et al., 1998). These observations highlight the role of the hippocampus in encoding relationships among environmental cues and in representing these relationships as percepts in the brain (O’Keefe and Nadel, 1978).

More conclusive evidence linking the hippocampus to spatial, contextual, and episodic memory came from studies involving a patient who, in 1953, suffered from severe and intractable epileptic seizures. Most first-year psychology textbooks refer to him as patient H.M., but after his passing in 2008, we now know him as Henry Gustav Molaison. To treat his condition, neurosurgeon Dr. William Beecher Scoville performed a bilateral temporal lobotomy. Following the removal of his hippocampus and adjacent structures, H.M. suffered severe anterograde amnesia; essentially the procedure rendered him unable to acquire new memories (Scoville and Milner, 2000). This demonstrated quite convincingly, the involvement of the hippocampus in forming episodic memories, that is, memory for unique personal experiences, (procedural memories for things like how to write or how to walk remained intact) and spatial memories as he was impaired on many spatial tasks (Corkin, 2002). The duality of function with respect to this brain area in processing both spatial and episodic memories is not surprising since episodic memory is spatiotemporal by definition.

Contextual memory is less easily defined than spatial or episodic memory. Contextual memory includes, but is not limited to the inclusion of spatial information and also relies heavily on the hippocampus (Holland and Bouton, 1999). It refers to the abstract components of experience providing meaning, placing events in time, encompassing perceptions, emotions, socially relevant information, and learned contingencies (Maren et al., 2013). Therefore, contextual information extends beyond spatial information to include other dimensions such as the physiological, motivational, social, and cognitive states of the organism. In the learning literature, contexts are distinguished from cues and typically refer to the set of circumstances surrounding an event or the physical location in which an event takes place. This suggests that contexts can be considered separate from the elements they encompass (Maren et al., 2013) and are yet still connected to them. Nadel and Willner (1980) describe context as being paradoxical for this reason (Anderson et al., 2003).

The hippocampus is involved in several memory processes including acquisition, consolidation, and retrieval (Bird and Burgess, 2008). For contextual learning to occur, a representation of the context must be formed or encoded in the hippocampus. A widespread hypothesis central to all neurobiological investigations of memory is the hypothesis that memory formation should result in a structural, observable memory trace (Hebb, 1949). Although this idea is often credited to Hebb (1949) postulate of memory residing in specific *cell assemblies*, this notion was inspired by Lorente de No (1933) reverberating *neural loops*, and is consistent with Semon (1921) idea of the *engram* to refer to these putative contextual memory traces, that we believe embody our experiences quite literally (Schacter et al., 1982). These ensembles can be tracked (Guzowski et al., 1999), tagged, and even artificially reactivated using optogenetics (Liu et al., 2013; Denny et al., 2014) in attempts to recapitulate experiences. Scientists have looked at the manipulation of two separate engrams simultaneously (Yokose et al., 2017) and have even been able to investigate how two distinct engrams formed at different times interact with each other (Won and Silva, 2008; Rogerson et al., 2014; Rashid et al., 2016; Grella et al., 2022). Today, we think of memory traces or engrams as the biochemical changes occurring following experience, set in a sparse population of neurons. These changes, which persist, involve the transcription of genes (Guzowski et al., 1999; Minatohara et al., 2016; Maple et al., 2017) and the formation of proteins. Moreover, these populations of neurons are reactivated when the memory is recalled (Guzowski et al., 1999; Reijmers et al., 2007; Han et al., 2009; Silva et al., 2009; Garner et al., 2012; Deng et al., 2013; Liu et al., 2013; Taylor et al., 2013; Ramirez et al., 2014; Rogerson et al., 2014).

For several decades scientists have been trying to study these traces debating about where they may be stored (Mayes and Roberts, 2001). The quintessential example being Lashley (1950) experiments, notably removing cortex portions in famously failed attempts to locate the engram. From this, he argued against the functional specialization and modularity of the brain, and concluded that memory must be distributed, but not redundantly. He expressed difficulty conceiving a satisfying mechanism but acknowledged that learning does occur. Indeed, it does. What Lashley could not envision was that the brain engages in a high degree of region specificity, yet there are multiple pathways to get to the same place. And despite his failed attempts, we now know that the hippocampus is a core brain structure supporting memory (Eichenbaum et al., 2012). But prior to any hard evidence that the hippocampus contained memory traces, Marr (1971) proposed, in his basic model of simple memory, that pyramidal cells within the hippocampus could be regarded as populations of cells in which simple representations of various input events are formed. He postulated that the hippocampus acts as a temporary storage space for sensory experiences that are encoded by specific patterns and that these patterns are retrieved when confronted with a cue. He also believed that this information would eventually be transferred to the neocortex. Given the lack of evidence at the time, and the astounding accuracy of his predictions, his theories were extremely prescient. Marr was also very much interested in computational modeling; thus, it is befitting that computational neuroscience models developed some 20 years later, would also predict that representations of the surrounding environment were formed in the hippocampus (Gluck and Myers, 1993; Treves and Rolls, 1994). We are now aware that the hippocampus does indeed form contextual representations of the surrounding environment (Hirsh, 1974; Fanselow, 2000; Guzowski et al., 2001; Rudy et al., 2004; Vazdarjanova and Guzowski, 2004). In fact, contexts can be robustly encoded very rapidly (<30 s) (Fanselow, 1986; Wiltgen et al., 2001). Moreover, lesions of the hippocampus impair contextual learning (Sutherland et al., 1982; Winocur and Gilbert, 1984; Selden et al., 1991; Kim and Fanselow, 1992; Phillips and LeDoux, 1992; Young et al., 1994; Chen et al., 1996; Gerlai, 1998).

The concept of a memory trace can seem very abstract. Our brief acquaintance with the material basis of memory (Tonegawa et al., 2015b) as engram cells has not included a specification of what information is encoded. For instance, an animal is placed in an environment and undergoes a unique experience (e.g., another mouse is placed in the box) and as a result, a distinct set of hippocampal neurons is recruited, the activity in which is considered to be a component of the distributed memory trace for that experience, encoding what we believe, is at least the contextual component of that episodic memory (Gerrard et al., 2001; Fyhn et al., 2007; Nalloor et al., 2012; McKenzie et al., 2013; Orsini et al., 2013; Takahashi, 2013; Cai et al., 2016; Kelemen and Fenton, 2016). But what does that mean–the contextual component? Different aspects of a memory may be encoded in different sub-regions. In the dorsal hippocampus, this includes spatial information given that neurons comprising these ensembles are spatially responsive (e.g., place cells), and traversing the environment, sampling its spatial properties, activates these cells (Chawla et al., 2005; Ramírez-Amaya et al., 2005; Vazdarjanova et al., 2006). Information related to the valence of a memory (i.e., positive or negative) may be encoded in the basolateral amygdala (BLA), social elements may be encoded in the CA2 (Alexander et al., 2016; Tzakis and Holahan, 2019), and emotional aspects of a memory in the ventral hippocampus (Fanselow and Dong, 2010; Shpokayte et al., 2022; Pronier et al., 2023). Therefore, the full context of an experience may only be recaptured when all of these populations are reactivated in concert, potentially increasing neural coding space to include many subregions (Holtmaat and Caroni, 2016).

Contextual information present at the time of memory encoding may be different than contextual information present at the time of retrieval. Tulving (1972, p. 352) described remembering as “the joint product of information stored in the past, and information present in the immediate cognitive environment of the rememberer.” This view emphasizes how memory can be affected by factors present at the time of retrieval (e.g., mood, hormones, vigilance, stress etc.), and that retrieval efficacy depends not only on the integrity of the memory trace alone, but also on these relevant contextual circumstances (Sara, 1985; Rimmele et al., 2016). Contextual cues play an important role in triggering or facilitating memory retrieval processes (Sara and Devauges, 1989). This is what Lashley failed to fully appreciate. In addition to the term engram, Semon (1921) also coined the term *ecphory* to describe the automatic process that occurs during memory retrieval between contextual elements and the memory traces they reactivate. Presentation of contextual cues before a test of memory retention can help to mitigate experimentally induced amnesia (Sara, 1974), natural time-dependent forgetting (Sara et al., 1980; Sara and Deweer, 1982), and memory deficits in rats with hippocampal lesions (Winocur and Kinsbourne, 1978). Contextual cues can also elicit changes in an individual through an influence on neurophysiological and attentional states and can even be conditioned to elicit biological changes that match the internal state of the individual during memory acquisition. The arousal experienced at the time of learning is essentially recapitulated in the brain during retrieval (Sterpenich et al., 2006) and can influence the retrieval process (Rimmele et al., 2016). One of Ivan Pavlov’s students, Pyotyr S. Kupalov first noticed this while studying conditioned behavior in dogs. He noticed that the strength of the dog’s conditioned response was greater when the dog was tested under the same conditions of illumination and noise that occurred during training (Giurgea, 1989) and posited that the context was able to affect his cortical tone such that this tone facilitated memory retrieval. He called this the *truncated conditioned reflex* (Sara, 1985). Other studies have replicated this finding in various circumstances to demonstrate that contextual information not only exerts a powerful influence over memory encoding and retrieval, but also over physiological responses that directly influence memory.

### 2.2 Spatially responsive neurons

A non-exhaustive list of spatially responsive neurons includes *head direction cells*, *place cells*, *grid cells*, *and boundary vector cells* (O’Keefe and Dostrovsky, 1971; Taube et al., 1990; O’Keefe and Burgess, 1996; Hafting et al., 2005). These cells, which can be found within the mammalian hippocampal formation, which in addition to the hippocampus include parahippocampal regions such as the subiculum, the presubiculum, and the entorhinal cortex (EC), and in a vital node that supports spatial learning, the anterior thalamus (TH). These cells are highly specialized. For instance, place cells are pyramidal neurons found predominately in the CA1/3, that fire when an animal visits a particular location in an environment (O’Keefe and Dostrovsky, 1971). The activity of these cells encodes the animal’s location in space, each cell with a different place field, with activity in local cell populations covering the rat’s entire environment (O’Keefe, 1976). Although recent evidence suggests some clustering of place cells in the CA1 (Wirtshafter and Disterhoft, 2023), generally speaking, these cells are organized in a manner where adjacent place cells do not necessarily give rise to adjacent place fields. In fact, neurons adjacent to a place cell are more likely to be silent within a given environment (Thompson and Best, 1989). This lack of topographical organization demonstrates that (1) not all hippocampal cells are place cells, (2) inputs are not topographically organized, and (3) the lack of firing may be just as important as the presence of firing.

This orchestration of activity and no-activity has been hypothesized to contribute to how hippocampal circuits synchronize frequencies (oscillations such as gamma and theta) (Thompson and Best, 1989; Mizumori, 2013) and may be related to hippocampal plasticity. Interestingly, place fields exhibit plasticity in that they can change when alterations in the environment occur. For instance, in one environment, a single place cell may become active when the animal visits the left corner of the room; in a different environment that same cell does not respond at all when the animal visits the left corner, and in a third environment, that cell becomes active in the right corner. This phenomenon in which place cells can alter their firing patterns in response to environmental changes was discovered by Muller and Kubie (1987) and is referred to as *remapping*. It is specifically this property that emphasizes the multi-representational nature of the hippocampus (Colgin et al., 2008). Place cells have been shown in numerous studies to remap in response to novel environmental stimuli, and although place cells can possess more than one place field within the same environment (Maurer et al., 2006), in some instances, they can also remap in the same environment as a result of experience (Navratilova et al., 2012). Most of the electrophysiology studies conducted to date have identified place cells in the CA1/3 region of the hippocampus, but there is some evidence for place cells in other brain regions such as the medial entorhinal cortex (mEC) (Quirk et al., 1992; Hargreaves et al., 2005; Savelli et al., 2008), the lateral septum (LS) (Nishijo et al., 1997; Zhou et al., 1999; Leutgeb and Mizumori, 2002), the TH (Jankowski et al., 2015), the retrosplenial cortex (RSC) (Tayler and Wiltgen, 2013; Cowansage et al., 2014), and the prefrontal cortex (PFC) (Zelikowsky et al., 2014). Cells in these regions have been identified as having *place-like* properties but typically have a much lower spatial resolution (Grieves and Jeffery, 2017). Several studies have also suggested that other types of neurons such as granule cells in the DG, which also encode spatial information, may function similarly to place cells (Tonegawa et al., 2015a). The formation of contextual representations is hypothetically driven by place cell activation, at least in rodents (Chawla et al., 2005; Ramírez-Amaya et al., 2005; Vazdarjanova et al., 2006; Rowland et al., 2011), which is coupled to the initiation of second messenger systems and gene transcription leading to protein synthesis (Miyashita et al., 2008).

Shortly after the discovery of place cells, it was determined that within the presubiculum there were cells that fired in response to the specific direction an animal’s head was pointed; cells had different preferences for different orientations (Taube et al., 1990) and these cells were called head-direction cells. Head direction cells have since been localized to other regions of the brain where the presubiculum projects to (e.g., TH, RSC) (Mizumori and Williams, 1993; Chen et al., 1994; Taube, 1995; Sherrill et al., 2013; Shine et al., 2016). The firing rates of both place, and head direction cells are often coupled, and to some degree controlled by an interaction between external landmarks and idiothetic cues (Yoganarasimha and Knierim, 2005), although further research is needed to characterize this interaction.

In layers II and III of the mEC there are cells that have multiple firing fields arranged in a tessellated, grid-like array that covers the surface of the environment, with firing occurring maximally for a cell when the animal is at any vertex of a grid of equilateral triangles (Hafting et al., 2005; Fyhn et al., 2007; Moser et al., 2008), hence, they were named grid cells. The grid fields tend to increase in size from the dorsal to ventral regions of the mEC (Hafting et al., 2005). In darkness, and when landmarks are removed, the cells maintain their fields suggesting they may play a prominent role in path integration, especially given that their fields are arranged in a way that could theoretically allow for vector algebra (Hafting et al., 2005; Barry et al., 2007).

Several studies have shown that place cells can predict an animal’s trajectory or goal location, demonstrating anticipatory properties of firing (Johnson and Redish, 2007; Schmidt and Redish, 2013), therefore, it is biologically plausible that grid fields contribute to the generation of place fields (Moser et al., 2008). Several theoretical models have emerged, inspired at least in part by the recent characterization of the bat hippocampus, hypothesizing the existence of a grid-like representation of space that includes the vertical as well as the horizontal plane since organisms travel in a three-dimensional environment (Jeffery et al., 2013). Three-dimensional representation of space by cells in the hippocampus has been examined in flying bats (Yartsev and Ulanovsky, 2013; Sarel et al., 2017). The place fields of place cells in the hippocampus of free-flying Egyptian fruit bats are spherical volumes (Yartsev and Ulanovsky, 2013), however, others theorize that these planes are processed separately (i.e., bi-coded system) (Phillips and Ogeil, 2013) while some predict that it depends on the direction the animal is moving (Jeffery et al., 2013). In deeper layers of the EC, conjunctive place and grid cells have also been found (Moser et al., 2008). In addition, a fourth type of neuron exhibiting a spatial profile has been discovered in the subiculum—boundary vector cells, also called border cells (Hartley et al., 2000; Lever et al., 2009). These cells are sensitive to geometric properties of the environment, specifically boundaries, and have also been proposed as inputs to place cells (Lever et al., 2009).

Learning more about how these neurons communicate with each other would give us a better understanding of how mammals represent space. It is believed that the collective firing of such cells, specifically place cells, within a given environment comprises the spatial or contextual code for that environment (Pevzner et al., 2012). Current models also provide evidence for the possibility of an associated temporal code (Moser et al., 2008). Certain cells in the CA1 and CA3 have been shown to fire at specific times during a task and have been dubbed *time-cells* (Colgin, 2016). Cells in the hippocampus exhibit a strong background low frequency (4–8 Hz) theta oscillation typically when an animal is engaged in active locomotion (e.g., exploration) or during REM sleep (Maurer and McNaughton, 2007), a rhythm that is entrained by the MS (Mizumori et al., 1989). To relate the timing of spikes to the theta frequency, each spike is assigned a phase (0–360 degrees, based on when it fires relative to the theta oscillation; 0 degrees corresponds to the trough of the oscillation). For a place cell that is anchored to a landmark, the phase can theoretically tell how far the landmark is, and whether the animal is entering or exiting that place field (Maurer et al., 2006; Moser et al., 2008). As the rat moves through a place field, toward or away from the landmark, the phase at which it fires changes from theta cycle to theta cycle and therefore spatial information is encoded in the timing of spikes for the theta rhythm (negative correlation of spike phase to animal position), a phenomenon called *phase precession* (Mehta et al., 2002). Neural activity across brain regions is thought to be synchronized by gamma (∼40 Hz) oscillations (Colgin and Moser, 2010), which occur in a phasic manner (grouped bursts) while theta oscillations occur in a tonic (ungrouped, stochastic) pattern (Bragin et al., 1995). Head direction cells may exert control over grid cells to aid in path integration. There is preliminary evidence to show that gamma oscillations can synchronize activity in different cell populations (Colgin and Moser, 2010) and may be linking head direction to place or grid cell activity in an associative *Hebbian* manner (Hebb, 1949). Ultimately, contextual information encoded as representations in the dorsal hippocampal and parahippocampal regions is multi-sensory, externally and internally driven, spatiotemporal information (Burgess et al., 2002).

## 3 Maintaining multiple contextual representations

### 3.1 Orthogonalizing output patterns and remapping

Contextual memory formation involves dynamic interactions across brain regions, e.g., the RSC encodes sensory input from allocentric frames of reference (Andersen, 1997; Colby and Goldberg, 1999; Parron and Save, 2004), the perirhinal cortex is involved in cue and object recognition (Mumby and Pinel, 1994), and the TH codes for visual and vestibular cues (Shine et al., 2016). That is, the integration of contextual information is supported by a neural architecture that is modularly connected within a system that is distributed with synchronous activation of different regions working together (Bennett, 1996; Bingman and Cheng, 2005; Cruse and Wehner, 2011). The DG and the CA3 work together to encode, store, and retrieve contextual memories. Consistent with Marr’s (Marr, 1971) description of the CA3 as part of an auto-associative network that can give rise to the recall or reconstruction of complete memories with only partial cues due to a relatively high level of interconnectivity (recurrent collaterals) (Colgin et al., 2008), it has been proposed that the CA3 engages in a process called *pattern completion* (Gluck and Myers, 1993; Treves and Rolls, 1994) necessary to form contextual representations by linking diverse inputs. This means that when simultaneous activities represent both the location and content of an episode, they are linked and preserved as a unified representation (Colgin et al., 2008). It may seem intuitive that the ability to reconstruct complete memories from only partial cues would promote interference, especially if operating on representations of slightly similar environments, but to potentially mitigate such interference, remapping is especially prominent in the CA3 (Colgin, 2016). Moreover, it has been shown that the CA3 utilizes a sparse coding scheme to form contextual representations and this is supported by lower levels of neuronal activity observed in the CA3 compared to the CA1, but a higher degree of overlap when comparing ensembles after visiting the same context repeatedly (Vazdarjanova and Guzowski, 2004). This is further supported by the fact that the CA3 seems to play an important role in one-trial learning; this region is extremely sensitive to environmental changes and can encode contexts very rapidly (Cravens et al., 2006; Miyashita et al., 2009).

*Pattern separation* is an opposing process that also serves to reduce memory interference and is associated with the DG. This is achieved by outputting highly dissimilar firing patterns, despite any similarity in input patterns (e.g., sensory input) (Deng et al., 2010). In other words, the DG is thought to act as a mediator of stimulus representations, which can perform stimulus discrimination while reducing interference from redundant stimuli by enhancing dissimilarity between representations (Leutgeb et al., 2007; Bakker et al., 2008; Berron et al., 2016; Kesner et al., 2016). The DG receives incoming spatial information via excitatory inputs from the EC and must process this information before sending excitatory outputs to the CA3. However, this structure is also under a high level of inhibitory control due to the presence of inhibitory (GABA-ergic) interneurons (e.g., basket cells) via feedback and feed-forward inhibition (Ribak, 1992; Jonas and Lisman, 2014). Moreover, the DG contains only a small fraction of neurons displaying activity at any given time. Therefore, low levels of basal activity combined with a vast number of granule cells (∼1 million neurons) contribute to a sparse, relatively orthogonal coding scheme that can support different traces assigned to different memories, promoting a reduction in interference from similar stimuli. For instance, if every year on Halloween your group of friends got together to watch a horror film, it may be difficult to remember which years you watched which films because these memories would have overlapping elements. This interference can hypothetically be overcome by employing a neural system that can maintain different representations for each experience, despite the similarity of these experiences (Colgin et al., 2008). The process of orthogonalizing output in contextual representations despite similarity in input is critical to this function (Gilbert et al., 2001; Chawla et al., 2005; Leutgeb et al., 2007). Thus, both the DG and the CA3 can engage in pronounced remapping (Leutgeb et al., 2004) and the sparse coding scheme used allows for similar events to be encoded by distinct populations of cells specifically to reduce interference. The generation of multiple contextual representations (global remapping) permits the distinction between similar experiences in different environments.

It is important to note though, that cells can also engage in *partial remapping* where only a subset of neurons remap, or *rate remapping* where maintenance of the same representation is preserved but the firing rate of the cells within that representation changes, theoretically to allow for differentiation of two different experiences in the same spatial context (Leutgeb et al., 2005). Neural mechanisms such as remapping are considered to be adaptive in an ever-changing environment, where animals must be able to update contextual representations to incorporate new information (Grella et al., 2019). For example, if an animal learns that a food reward is present in a certain location within an environment, this information would theoretically be encoded within a specific neuronal ensemble. If the next day, the reward is no longer present in that location, but has moved to a different location, then the animal would need to update the representation associated with this experience and this could be achieved via a remapping effect. While significant advances have been made in the last 50 years toward a better understanding of how representations of the surrounding environment are created and stored, the way in which these representations change and are modulated is not fully understood.

To appreciate how contextual representations, change or remap globally, we must consider that sensory input is relayed to the DG/CA3 via the EC and that the mEC is also where grid cells reside. Therefore, it is plausible that grid cells contribute to global remapping. One piece of evidence supporting this hypothesis is the finding that changes in the environment that induce rate remapping in the CA3 do not shift grid cell representations whereas changes that induce global remapping in the CA3 not only cause grid cells to shift, but do so in a temporally synchronous manner (Fyhn et al., 2007; Colgin et al., 2008). Moreover, partial inactivation of the mEC causes remapping in the CA3 (Bergado et al., 2007; Miao et al., 2015). Another possibility is that changes in contextual representations are initiated through perturbations in hippocampal theta rhythm, which depend on projections from the MS. The MS may induce synaptic changes in the mEC, which are then relayed to place cells in the DG/CA3 resulting in remapping. This may be achieved via cholinergic inputs from the MS to the DG (Bergado et al., 2007). Finally, a third possibility involves a direct projection from the EC to the CA1 that acts in parallel to the traditional trisynaptic pathway (EC → DG → CA3 → CA1) which posits the CA1 as a novelty/prediction error detector (Basu and Siegelbaum, 2015) that compares stored representations in the DG/CA3 with ongoing, direct sensory inputs from the EC. The CA1 sends excitatory projections to layer V of the EC, which in turn, loops back to layers II and III (Naber et al., 2001). This hypothesis suggests that through this connection, the CA1 translates the detected prediction error into a signal, which recruits new cells in the DG/CA3 to become active (Lee et al., 2005; Chen et al., 2011; Duncan et al., 2012). Interestingly, acute inactivation of the mEC induced remapping in the hippocampus (Miao et al., 2015) while bilateral excitotoxic lesions of the mEC had no effect, or could not abolish remapping (Schlesiger et al., 2015) therefore, it is unclear what role the mEC or grid cells play in global remapping and further research is warranted. The complexity of this system is extended when you take into consideration that contextual representations may be externally or internally driven (Pastalkova et al., 2008). Furthermore, the fields of these spatiotemporal cells may be biased by sensory information (Ranck, 1973; O’Keefe, 1976; O’Keefe and Conway, 1978; Olton et al., 1978; Muller and Kubie, 1987; Gothard et al., 1996; O’Keefe and Burgess, 1996; Wiener, 1996; McEchron and Disterhoft, 1999; Save et al., 2000), task demands (Markus et al., 1995; Wood et al., 2000; Smith and Mizumori, 2006; Satvat et al., 2011), or motivational states (Breese et al., 1989; Kobayashi et al., 1997; Fyhn et al., 2002; Hölscher et al., 2003; Tabuchi et al., 2003; Kennedy and Shapiro, 2009). Mechanisms such as remapping are likely involved in the updating of memories and new learning, aiding animals in adapting to changing environments by updating contextual representations, but the precise modulation of these representations requires further investigation.

### 3.2 Temporal dynamics of immediate early gene transcription

One of the first steps in long-term plasticity is the transcription of immediate early genes (IEGs) such as *arc* (Activity Regulated Cytoskeletal-Associated Protein) also known as *arg3.1* (Link et al., 1995), and *zif268* also known as *egr1* (Guzowski, 2002). Unlike most genes, these genes do not require *de novo* protein synthesis to be transcribed as constitutive regulatory transcription factors (RTFs) such as cAMP response element binding protein (CREB) and serum-response factor (SRF) are available in the nucleus and capable of recruiting transcriptional machinery (Ginty, 1997; Finkbeiner and Greenberg, 1998). RTFs are activated by second messengers such as protein kinase A (PKA), calcium and calmodulin-dependent kinase IV (CaMK-IV), and mitogen-activated protein kinase (MAPK) following *N*-methyl-D-aspartate (NMDA) receptor-mediated synaptic stimulation.

Immediate early gene transcription occurs at low levels under basal conditions (Hargreaves et al., 2005; Miyashita et al., 2008) and is highly dependent on synaptic input (Lyford et al., 1995) although once initiated, can occur extremely rapidly (Cole et al., 1989; Guzowski et al., 1999; Vazdarjanova et al., 2002). Some IEGs regulate the transcription of other genes (RTFs) (e.g., *zif268*) and may play a role in metaplasticity (Guzowski, 2002; Maple et al., 2017), and other non-RTF IEGs (called effector IEGs) such as *arc*, are involved in a wide range of cellular functions (Miyashita et al., 2008). Suggestive of a highly specific function (Miyashita et al., 2008) *arc* is only found in vertebrates (Link et al., 1995; Lyford et al., 1995; Mattaliano et al., 2007) and is thought to promote plasticity via synaptic modifications (Rial Verde et al., 2006) such as the scaling/trafficking of α-amino-3-hydroxy-5-methyl-4-isoxazolepropionic acid (AMPA) receptors which mediate neuronal transmission (Xiao et al., 2000; Chowdhury et al., 2006) and initiating changes in the actin cytoskeleton of the cell required for changes in dendritic spine structure and density (Dillon and Goda, 2005). Following transcription, *arc* mRNA is rapidly transported outside of the nucleus to the dendrites for local storage, translation, and decay (Steward et al., 1998). *Arc* is one of the most tightly regulated proteins (Bramham et al., 2010) with a half-life of only 47 min (Hargreaves et al., 2005).

Consequently, IEGs have been widely used as neuronal markers of activity and due to the kinetics of IEG mRNA following transcription they can be used to map the activity history of individual neurons (Guzowski et al., 1999). A sensitive molecular protocol referred to as cellular compartmental analysis of temporal fluorescent *in situ* hybridization (catFISH) allows for the tracking of neuronal populations at two distinct time points by exploiting the distribution dynamics of IEG transcription. Following neuronal stimulation, the induction of *arc* mRNA occurs in the nucleus; these transcripts then translocate to the cytoplasm after approximately 15 min targeting the dendrites and returning to basal levels after approximately 60 min (Guzowski et al., 1999). In experiments utilizing this protocol, animals are typically placed in an environment that they are permitted to explore thus activating place cells, which drives *arc* transcription. After 5 min of context exploration, animals are placed back in their home cage, where any further transcription can be attributed to, and is associated with, the context that was just explored (Marrone et al., 2008). Twenty-five minutes later animals are given another context exposure for 5 min. Given the distribution dynamics of *arc* transcription, cells active during the second exploration will still contain *arc* mRNA in the nucleus but those cells, which were active during the first context exploration, will contain *arc* mRNA in the cytoplasm, and cells that were active in both behavioral epochs, will contain *arc* in both locations. Therefore, the sub-cellular localization of *arc* visualized via fluorescent confocal microscopy allows for the neuronal populations activated by two distinct experiences to be discriminated and quantified (Guzowski et al., 1999).

The catFISH protocol, developed by Guzowski et al. (1999), allows us to look at large numbers of cells, within many different brain regions simultaneously. Furthermore, it has demonstrated that *arc* expression is induced in the CA1 in a context-dependent manner. When animals visit the same context twice, as opposed to two different contexts, this results in a higher degree of overlap in the cells being activated across time points. This effect does not disappear or habituate following repeated context presentations across days and only after four exposures to the same context each separated by 25 min does *arc* induction begin to diminish (Hernandez and Abel, 2008). However, when the animal is presented with a new environment, even after nine exposures to the same context, this attenuation in *arc* transcription is rescued. The fact that *arc* induction is not easily disengaged when an animal is presented with familiar stimuli suggests that it does not distinguish between new learning and memory retrieval (Guzowski et al., 2006; Miyashita et al., 2008). This effect is also consistent with electrophysiology studies involving place cell remapping. Remapping occurs when an animal visits two different contexts in the same way different neuronal ensembles are recruited to activate *arc* in different contexts using the catFISH protocol. The tracking of IEGs in a temporal fashion has also been useful in determining the differential contributions of distinct subfields within the hippocampus to contextual coding. For instance, novel contexts appear to be encoded more rapidly in the CA3 compared to the CA1 (Guzowski et al., 2001; Pevzner et al., 2012) and spatially selective IEG expression has been demonstrated in the CA1 (Guzowski et al., 2006), the CA3 (Vazdarjanova and Guzowski, 2004) and the DG (Marrone et al., 2011; Schmidt et al., 2012). This technique is useful for exploring how contextual representations may potentially overlap across contexts. An interesting question specifically in the context of attractor neural network computational modeling, is how much overlap is necessary to denote some association while still preserving memories as distinct (Gastaldi et al., 2021). The answer may depend on the method used.

### 3.3 Immunohistochemistry to detect overlapping memory traces

While the catFISH method tracks mRNA expression in specific neurons over time to determine if a neuron was active in distinct or overlapping memory traces, immunohistochemistry, a widely adopted immunostaining technique, provides similar insights by employing antibodies to identify antigens corresponding to IEG products and other cellular proteins that serve as markers of neuronal activity. For example, by employing activity-dependent neuronal tagging techniques like Tet expression systems regulated by doxycycline, it becomes possible to genetically modify neurons that become active in response to a specific stimulus (Reijmers et al., 2007). These modified neurons express IEGs, and the promoters of these genes are used to drive the subsequent activation of fluorescent markers, which can be observed under a microscope for visualization. These systems offer the flexibility of creating *tagging windows*, allowing the marking of an initial memory trace. Later on, the organism can undergo another experience, and through immunohistological methods, the detection of IEG protein products can reveal overlaps between the two sets of markers (Grella et al., 2022). This information can provide insights into whether a particular neuron was involved in the formation of the initial memory trace, the second memory trace, or both.

### 3.4 *In vivo* calcium imaging to detect overlapping memory traces

More recently, researchers have also used genetically encoded calcium indicators (GECIs) such as GCaMP to serve as a valuable tool for tracking neuronal activity (Oh et al., 2019). GCaMP is introduced into specific neurons, either via transgenic organisms or viral vectors and once expressed, GCaMP undergoes a conformational change in response to increased intracellular calcium levels, resulting in a measurable fluorescence signal. Fluorescence microscopy, particularly two-photon microscopy, enables real-time imaging of the GCaMP-expressing neurons, providing high-resolution insights into individual neurons or targeted populations. In *in vivo* experiments, GCaMP allows for the dynamic observation of neuronal responses within a living organism, capturing temporal dynamics associated with physiological or behavioral conditions. In the study of overlapping engrams using *in vivo* calcium imaging, researchers can selectively mark specific populations of neurons associated with distinct memories or experiences, capturing the temporal and spatial patterns of neuronal activity during memory encoding and retrieval (Zaki et al., 2022). The analysis focuses on identifying overlapping engrams by examining whether the same neurons or neuronal populations are activated during the recall of multiple memories. Computational tools and statistical methods are then applied to process the imaging data, quantify the degree of overlap, and provide insights into the neural mechanisms underlying the representation of related memories in the brain. This integrated approach offers a dynamic perspective on the plasticity and shared neuronal substrates associated with memory encoding and retrieval.

## 4 Locus coeruleus hippocampal pathway in memory updating

### 4.1 Memory malleability: memories can and do change over time

By virtue of Hebbian plasticity, memories are malleable, an adaptive mechanism that allows an organism to always have access to the most relevant information in memory (Ye et al., 2020). From a functional perspective, this type of memory system allows for new information to be incorporated into a memory trace and for that trace to be *updated*. Why are memories not fixed so that you always remember events as perfectly as they occurred in real-time? The functional significance of a system where memories can be modified is highly debated and may even seem maladaptive in some cases (Rodriguez-Ortiz and Bermúdez-Rattoni, 2007). For instance, encoding can be distorted in such a way that elaborates certain semantic details of an event to achieve a sense of coherence (Fairfield et al., 2016). For this reason, eyewitness testimonies can be unreliable (Bartlett, 1932; Loftus and Palmer, 1974); people fill in gaps with imagined elements to create a complete picture in their minds. In these instances, especially if an individual is experiencing a highly emotional state, focus tends to be on the emotional content rather than the neutral contextual details (Fairfield et al., 2016). Although the flexibility of memories may not bode well in a courtroom, this sort of memory modulation can potentially have survival value in many other contexts and memory doesn’t always need to be accurate to be adaptive. For instance, fear learning and even the generalization of fear memories, has clear potential to be adaptive: you don’t need to know which rattlesnake tried to bite you, but the sound is enough to serve as a warning signal to keep you away. However, when fearful memories such as those acquired after experiencing a traumatic event become strengthened in a maladaptive way, this can lead to disorders such as post-traumatic stress disorder (PTSD). Despite our proclivity to view memory as an accurate depiction of past events like photos on your phone (Lee et al., 2017), mnemonic processes do not operate like a recorder that can be played back later for review.

Likewise, memory is commonly thought to progress linearly through stages such as encoding, storage, and retrieval, with the belief that memories stabilize over time through *consolidation* (Miyashita et al., 2009). Consolidation depends on *de novo* protein synthesis as inhibitors of protein synthesis disrupt late-phase long-term potentiation (LTP) thus interfering with the expression of long-term memory (Flexner et al., 1963; Agranoff et al., 1965; Davis and Squire, 1984; Krug et al., 1984; Goelet et al., 1986; Frey et al., 1988; Hernandez and Abel, 2008). Despite the organized description of memory, it’s crucial to note that memory is a complex and highly malleable construct, challenging the notions of unity and linearity. And contrary to the long-standing belief that memories were static or inflexible, recent understanding highlights their dynamic nature (Otis et al., 2015). Memories, when retrieved, undergo a process known as *reconsolidation*, rendering the reactivated memory trace temporarily susceptible to modification (Nader, 2015). This dynamic reconstruction can lead to the strengthening, weakening, or alteration of the original memory, with new contextual elements potentially overwriting it (Rimmele et al., 2016). Various factors during retrieval, such as internal motivation, hormonal profile, emotional state, and attention level, influence the reconsolidation process. In this context, we investigate the role of catecholamines, specifically norepinephrine (NE) and dopamine (DA), in hippocampal-dependent memory. Our exploration aims to elucidate a potentially significant pathway for memory updating, highlighting the involvement of NE/DA projections from the locus coeruleus (LC) to the hippocampus in the remapping and sculpting of contextual representations (Grella et al., 2019, 2021).

### 4.2 Catecholamines as neuromodulators of memory

The release of catecholamines throughout the mammalian brain is important for modulating attention, arousal, stress responses, and cognition as well as regulating hippocampal function (Hagena et al., 2016) and is likely involved in updating memories (Grella et al., 2019, 2021). Structurally, both NE and DA are quite similar. NE is generated by the amino acid tyrosine, which is converted to L-DOPA via tyrosine hydroxylase. Using AADAC, L-DOPA is transformed into DA. DA is transformed into NE via dopamine- β-hydroxylase (Smeets and González, 2000). NE exerts its effects by binding to adrenoreceptors (ARs). Pharmacodynamically, ARs are G-protein coupled receptors (GPCRs) and include α and β-subtypes (Ahlquist, 1948; Ramos and Arnsten, 2007) categorized by their affinity for NE (Lands et al., 1967). NE has different effects depending on the target receptor that is activated (Foote et al., 1983; Berridge and Waterhouse, 2003). Found mostly postsynaptically, α1 receptors are coupled to the guanine nucleotide-binding regulatory protein Gq, and when activated, this causes an increase in intracellular levels of Ca^2+^, and subsequently the release of NE (Perez, 2007). Conversely, α2 receptors are mostly presynaptic, coupled to Gi; activation of these receptors inhibits NE release acting as a negative feedback mechanism (Abercrombie et al., 1988), while α2 antagonists increase NE transmission (Abercrombie et al., 1988; Thomas and Holman, 1991; Hein et al., 1999). β-receptors (Mueller and Fagan, 2008; Nathan et al., 2008; Paul et al., 2009) are found pre and post-synaptic, positively coupled to Gs, which increase NE release when activated (Lands et al., 1967; Rogawski and Aghajanian, 1982; Misu and Kubo, 1986; Stein et al., 1993; Waterhouse et al., 1998; Benarroch, 2009). In humans, presynaptic β-receptors alter local release of NE (Stein et al., 1993). All subregions of the hippocampus contain adrenoreceptors (α1, α2, β 1, and β 2) and the LC is the only source of NE for the hippocampus (Moore and Bloom, 1979; Aston-Jones et al., 2004; Borges et al., 2017). Outside of neurogenesis, which is regulated by α1ARs (Perez, 2007), β-receptors are most commonly linked with hippocampal synaptic plasticity (Harley, 2007; Gelinas et al., 2008; Kemp and Manahan-Vaughan, 2008) with the DG containing the highest concentration of receptors (Hansen and Manahan-Vaughan, 2015), as well as the highest fiber density of LC-hippocampus projections and therefore, the highest levels of NE release occurring here (Moore and Bloom, 1979; Loy et al., 1980; Hansen and Manahan-Vaughan, 2015).

Generally, NE release is associated with increased heart rate and blood glucose levels. In preparation for fleeing or fighting, activation of the sympathetic nervous system and the release of catecholamines such as NE, occurs in mammals when faced with a threatening situation. Washburn and Cannon (1917) coined the term *fight or flight* to describe this hyperarousal reaction. Kety (1970) introduced the idea that biogenic amines such as NE, not only have an effect on arousal, but also on emotion and learning acting as neuromodulators. Neuromodulation is often contrasted with fast synaptic transmission where transmission is slow-acting on GPCRs rather than fast-acting on ligand-gated ion channels. Rather than initiating spiking, effects can be to modulate ongoing spiking activity, are typically long-lasting, and groups of cells are affected as opposed to one or two cells. Evolutionarily, neuromodulators emerged quite early and have been highly conserved. For instance, both DA and acetylcholine are present in invertebrate species of animals, however, NE is unique in that it is only present in vertebrates. Since Vittorio Erspamer discovered the biogenic amine octopamine in the salivary gland of an octopus and characterized it as having NE-like properties affecting physiology and behavior, it is thought that this chemical is the precursor to NE (Nair et al., 2019). The similarity between NE and octopamine demonstrates the conservation and need for such a molecule. Kety (1970) hypothesis regarding NE was quite specific; he believed that forebrain NE acted to selectively enhance cell firing in neurons receiving inputs during affectively important events and that this served to promote memory. He asserted that “The state of arousal by means of adrenergic input to each (cerebral, hippocampal, and cerebellar cortices) may serve to concurrently reinforce and to consolidate the significant sensory patterns, the affective associations and the motor programs necessary in the learning of a new adaptive response” (Kety, 1970).

Kety (1970) ideas were quite novel given the limited evidence at the time, demonstrating that neuromodulators could affect more than simply neuronal responses, but could also improve cognitive performance. In the brain, NE is produced in the LC, a small pontine cluster. This bilateral structure contains approximately 1600 densely packed neurons per nuclei in the rodent brain, all of which produce NE, all of which provide a neuromodulatory influence in the brain. The survival of an organism depends highly on its ability to remember certain information; therefore, it is adaptive for that organism to possess a mechanism by which it can detect what is important, highlight that information, and filter out what is irrelevant (Berridge, 2008). Kety (1970) believed that catecholamines played this role. The ability to demonstrate an insensitivity to the environment when rest is required, have a broad *diversive* focus in a manner of reconnaissance exploration when searching for resources, and maintain an *inspective* vigilant hold on an identified predator, food source, or potential mate when necessary, is key (Berlyne, 1966; Flicker and Geyer, 1982). This great task of processing such a wide variety of stimuli in a constantly changing environment is achieved by one of the smallest nuclei in the brain, the LC (Schwarz and Luo, 2015).

LC activity depends on the state of the animal (Bouret and Sara, 2005; Benarroch, 2009) with firing characterized as either *tonic* or *phasic* (Aston-Jones and Cohen, 2005; Berridge, 2008; Sara, 2009). Tonic firing consists of a sustained and regular pattern which is commonly associated with wakefulness, with firing decreasing during low arousal (e.g., sleep) and events such as eating and grooming (Aston-Jones and Bloom, 1981; Rajkowski et al., 1994; Grant et al., 1998). This firing pattern is also observed in response to changes in behavioral states such as stress (Aston-Jones and Cohen, 2005). When tonic firing increases, both attention and phasic activation decrease (Aston-Jones and Bloom, 1981; Rasmussen et al., 1986; Aston-Jones et al., 1999). In contrast, during tasks that require more focused attention for accurate task performance, or when an animal is exposed to novel or arousing stimuli, phasic burst firing is observed (Aston-Jones and Bloom, 1981; Sara and Segal, 1991; Tse et al., 2023). Phasic LC discharge has also been associated with the presence of salient stimuli (Aston-Jones et al., 1999), for instance, those that signal the availability of reward (Rajkowski et al., 1994) and can come to predict reward. The release of NE within the hippocampus depends on these patterns of firing (Harley, 1991).

### 4.3 Hippocampal catecholamine release, plasticity, and remapping

Both phasic and tonic LC activity can induce downstream plasticity effects. For instance, LC activation can increase NE release in the DG (Dahl and Winson, 1985; Harley and Milway, 1986; Harley et al., 1989; Babstock and Harley, 1992; Frizzell and Harley, 1994; Klukowski and Harley, 1994; Walling et al., 2004; Lemon et al., 2009) and CA1 (Lemon et al., 2009), which can subsequently lead to enhancements in LTP (Bliss et al., 1983; Neuman and Harley, 1983; Gray and Johnston, 1987; Hopkins and Johnston, 1988; Harley, 1991; Walling et al., 2004, 2011; Almaguer-Melian et al., 2005; Lashgari et al., 2008; Lim et al., 2010; Hagena et al., 2016) and long-term depression (LTD) (Lemon et al., 2009; Lemon and Manahan-Vaughan, 2012; Hansen and Manahan-Vaughan, 2015). Elevated levels of NE can increase somatic and dendritic excitability in the DG (Lacaille and Harley, 1985; Stanton and Sarvey, 1985; Harley, 1991; Hagena et al., 2016) as well as in CA1 and CA3 (Mueller et al., 1981; Heginbotham and Dunwiddie, 1991; Dunwiddie et al., 1992; Jurgens et al., 2005a,b), effects which are mediated by βARs (Perreault et al., 2014) and which can persist for 24 h (Walling and Harley, 2004). Administration of nisoxetine (NE reuptake inhibitor), or idazoxan (α2 adrenoceptor antagonist) can enhance LTP while clonidine (α2 adrenoceptor agonist) can impair LTP (Lim et al., 2010). Therefore, it has been proposed that activation of the LC-NE system can induce changes in network dynamics occurring at critical times when learning is necessary to promote adaptive behavior (Sara et al., 1994; Bouret and Sara, 2005).

Using high performance liquid chromatography to analyze hippocampal NE/DA release following optogenetic stimulation of the LC, both NE and DA were increased by 400% during light-on conditions, with a NE/DA ratio of 1:10 (Kempadoo et al., 2016). Like NE, DA release within the hippocampus has also been demonstrated to play a neuromodulatory role on memory (Cropley et al., 2006; Mehta and Riedel, 2006; Kempadoo et al., 2016) and has been shown to enhance novel information coding through hippocampal synaptic changes (Lemon and Manahan-Vaughan, 2012). Phasic LC activation has been shown to potentiate LTP at CA3-CA1 synapses, which is blocked with application of the DA antagonist SCH23390 (Takeuchi et al., 2013) demonstrating a role for DA in initiating hippocampal plasticity. In the context of reinforcement learning, DA is classically considered to be a learning signal coding reward prediction errors, released during times of uncertainty (Diederen and Fletcher, 2021). The hippocampus is hypothesized to act as a *detector* of prediction errors (Basu and Siegelbaum, 2015). That is, the hippocampus stores contextual representations and defines the expectations for these contexts. For example, you have a representation stored of the coffee shop down the street. You venture out to get coffee one morning, and you find the door is locked, when it is usually open at this time of day. When the current experience does not match the expected, this results in a context prediction error (Mizumori, 2013). An example of this in a research setting involves fear conditioning. Animals that have been fear-conditioned in a particular context form a representation of that context. Upon re-exposure to the context the following day, these animals *predict* that they will again receive a shock in that environment, but during this second session, this does not occur. In the first context presentation, given the association between the shock and the context, the animal learns to fear the context. During the second context presentation, despite that the context itself is the same, the absence of the shock suggests that the context is in fact, safe. Identification of mismatches provides a signal that a new representation is needed, or that the old representation needs updating. This process allows for the distinction of memories into separate, meaningful epochs (Mizumori, 2013) and most importantly, allows for learning to occur. And indeed, representations do change as a result of different stages of learning (Wang et al., 2012). What drives this change? What provides the substrate for new learning in these situations? And how do these changes manifest at the cellular level?

Given the role of the hippocampus as a mismatch detector, and the co-release of DA with NE in the presence of salient information when memory updating is required, it is not surprising that DA may have a similar role in the hippocampus as it does in the mesolimbic and mesocortical DA systems. DA, the catecholamine precursor to NE (Smeets and González, 2000) also binds to GPCRs found within the hippocampus. There are two subfamilies of DA receptors: D1-like (D1 and D5) and D2 (D2, D3, and D4) (Jaber et al., 1996; El-Ghundi et al., 2007). Previous studies show that the ventral tegmental area (VTA) (site of DA synthesis) participates in the regulation of protein synthesis in memory consolidation via D1/D5 activity. Moncada (2017) suggested that the LC-DA system may have a comparable role, operating independently and complementarily to the VTA. More specifically, he proposed that both D1/D5 and βARs are necessary for the synthesis of plasticity related proteins, which play a critical role in memory consolidation. This aligns with earlier research demonstrating that infusions of SCH23390 abolishes the enhancing effect of introducing animals to spatial novelty in the transition from early to late LTP in the CA1 (Li et al., 2003). It was also recently found that dorsal LC CA1 fibers modulate memory updating through both NE and DA release (Gálvez-Márquez et al., 2022). More specifically, researchers found that both NE and DA modulation in the dorsal CA1 are necessary for the behavioral expression of new learning in an object location task, but that only LC-DA is required to update spatial contextual recognition memory. The exact role of DA in the hippocampus and its interplay with LC-DA in terms of plasticity effects is still not well-understood. We anticipate that future studies will likely concentrate on unraveling these mechanisms, particularly in the context of memory updating. It is possible that these brief neuromodulatory signals are coded as novelty/salience in the sense that both NE and DA can guide attention toward a stimulus because it is novel, and that this provides the substrate for new learning (Lisman and Grace, 2005; McNamara et al., 2014). For instance, Kempadoo et al. (2016) found that selective attention toward a stimulus was enhanced, as well as spatial recognition, following DA release acting on D1/D5 receptors in the dorsal CA1 following optical activation of the LC. While LC NE activation may serve as a novelty signal involved in updating contextual representations, promoting cognitive flexibility by biasing memory systems toward encoding novel hippocampal sequences, DA may be specifically encoding the salience or value of that stimulus and gating plasticity by modulating its influence on memory (i.e., affecting subsequent models and predictions as would be the case in the presence of a prediction error). It has been recently shown using *in vivo* calcium imaging that optical stimulation of LC neurons can induce plasticity in the visual system within minutes, simulating learning at a highly increased speed that would normally take place over days. The authors of this study concluded that prediction errors drive LC activity to achieve this plasticity (Jordan and Keller, 2023).

In terms of memory updating and plasticity effects involving remapping in the hippocampus, earlier work has shown that phasic activation of the LC led to remapping of contextual representations in the CA1/CA3 and DG (Grella et al., 2019). Animals experienced either repeated exploration of the same context or exploration of two different contexts. Rats exposed to two distinct contexts exhibited separate representations, while those reintroduced to the same context reactivated the original engram. Intriguingly, phasic, but not tonic, activation of the LC prompted a reset of this engram, even in the absence of a physical change in the context. In this study, it was not determined if these effects were noradrenergic or dopaminergic (i.e., they did not try to block these effects with the βAR antagonist propranolol or SCH23390). Nevertheless, this work is consistent with the idea that the LC supports and modulates hippocampal-dependent memory playing a pivotal role in shaping our memories through catecholaminergic modulation leading to subsequent plasticity effects on both memory encoding and subsequent consolidation. Interestingly, one study also found that silencing the LC using DREADDs also resulted in place cell remapping in the CA3 (Wagatsuma et al., 2018). During spatial learning, place cell remapping can accommodate new goal locations, however, there is not an abundance of information regarding the underlying neurocircuitry supporting this phenomenon Segal and Bloom (1976) showed in rats, that electrical LC stimulation selectively enhanced hippocampal responses to stimuli conditioned with an appetitive reward. Using two-photon calcium imaging Kaufman et al. (2020), showed that projections from the LC to the CA1 may be involved in signaling the relocation of a reward in a goal-oriented spatial learning task in a manner that allowed the researchers to predict behavioral performance. They also found that optical stimulation of the LC induced place cell remapping in the CA1 whereas inhibiting this pathway prevented this reorganization. These studies offer insights into the impact of neuromodulatory actions on hippocampal contextual representations and plasticity surrounding the stability and flexibility of these engrams.

### 4.4 Hippocampal catecholamine release and memory encoding

Over the years, many studies have demonstrated that catecholamines can affect different aspects of memory (Grella et al., 2021). There have been inconsistencies in these results, which may be the result of differences in methodology. Differentiating whether an experimental manipulation exerts its effects on memory encoding vs. memory consolidation is not easy. With the use of neuroimaging techniques, it may be possible to assess these differences in real time. Studies that do not involve neuroimaging require temporal considerations such that manipulations are applied immediately before or during learning, which may impact encoding, in comparison with manipulations applied after a delay, which may affect consolidation. Another thing to consider is how long after learning memory retention is measured. Thus, far fewer studies have examined the role of catecholamines on memory encoding compared to consolidation.

Hagena et al. (2016) suggests that the encoding of novel stimuli involves βAR activation. Evidence for this comes from the fact that lesioning the LC, which disrupts the transmission of NE to the hippocampus, impairs the acquisition of spatial learning (Anlezark et al., 1973; Compton et al., 1995; Coradazzi et al., 2016) with a greater effect following bilateral lesions (Compton et al., 1995). The LC innervates the CA3 more densely than other subregions of the hippocampus. This may be related to the involvement of the CA3 in single-trial learning of novel experiences and the crucial neuromodulatory role of the LC in quickly forming stable engrams in the CA3 (Wagatsuma et al., 2018). However, it is worth noting that clonidine injections in the LC did not impair performance in a delayed non-match to position radial arm maze task, although this task measures spatial working memory, a function that also engages the PFC (Mair et al., 2005). Similarly, there was no impact on performance in a T-maze spatial task despite bilateral LC lesions, which resulted in a significant decrease (67–90%) in cortical NE levels (Amaral and Foss, 1975).

In a previous study, isoproterenol (βAR agonist), was infused into the DG immediately prior to a reversal learning task involving reference memory in a Barnes maze (Grella et al., 2021). This type of learning requires encoding new contextual maps. Initially, this manipulation resulted in a decrease in latency to find the new correct escape location, which would imply an impairment. However, these animals were tested on retention of this memory during a probe test several days later, and they demonstrated enhanced performance compared to the other groups including a group that received propranolol as well as isoproterenol. Thus, labeling this as an impairment is subject to interpretation and really depends on the stage of training memory is measured. DA antagonists were not administered in this study. These findings suggest that NE release in the hippocampus can act as a neuromodulatory switch to bias memory encoding over retrieval, an effect which may appear maladaptive in the short term and adaptive in the long term (Grella et al., 2021). Consistent with this theory of LC neuromodulation of memory traces, activation of the LC and subsequent NE release, facilitated encoding of a spatial memory via βARs (Lemon et al., 2009). Additionally, lidocaine-induced inactivation of the LC immediately after, but not 90 or 360 min after, impaired inhibitory avoidance learning suggesting that LC-NE may be critical to encoding this type of learning (Wilson and McNaughton, 1994).

*Encoding*, the first step in memory formation, involves the recruitment of engram cells at the time of learning. What determines which cells will be recruited? Recent studies have shown that neurons compete for the allocation of engrams and those more likely to be allocated also show higher function of the transcription factor CREB (Park et al., 2016, 2020). Thus, neurons are selectively engaged in memory encoding owing to their distinct intrinsic characteristics. Supporting this idea, the intrinsic cellular excitability, representing the likelihood of a neuron to generate an action potential in response to input, emerges as a crucial factor influencing their involvement in memory processes (Silva et al., 2009). Catecholamines released in the hippocampus via the LC may be able to bias memory systems toward encoding through CREB signaling pathways. Moreover, novelty and stress, both associated with higher levels of NE release, have been shown in several cases to facilitate memory encoding (Tulving and Kroll, 1995) and are associated with CREB activation and the upregulation of CREB-regulated genes such as brain-derived neurotropic factor (BDNF) (Kabitzke et al., 2011). Optogenetic activation of the LC has been shown to mimic novelty, both when applied prior to, and after encoding (Tse et al., 2023); an effect that was blocked with SCH23390. Additionally, in the CA1, DA has been shown to modulate intrinsic neuronal excitability via D1/D5 receptors (Edelmann and Lessmann, 2013). While the role of astrocytes in learning and memory has been well established (Suzuki et al., 2011), the idea of astrocytic ensembles has received less attention (Delgado and Navarrete, 2023) and the role of astrocytes in memory allocation has not been delineated. However, it has been shown that NE activates CREB in cortical astrocytes (Carriba et al., 2012) and increased BDNF levels are observed following application of NE, DA, and selective α1 and βAR agonists (Koppel et al., 2018).

Given that neuronal excitability is a determinant in the allocation of cells that make up a particular engram (Silva et al., 2009), it is also a factor in encoding an event that occurs shortly after (Cai et al., 2016), thus implicating the LC in memory linking (Chowdhury et al., 2022). *Memory storage* refers to the maintenance and preservation of memories (Squire, 2009) whereby flexible synaptic connections reshaped by learning serve as essential components in this process (Ryan et al., 2015). The idea that memories may be stored as *synaptic weights* has a long history. For instance, Jones (1994) hypothesized that enhancement in synaptic efficacy could be a mechanism of memory storage. However, it is important to note that simple enhancement in synaptic efficacy is not sufficient to store a complex memory but that these changes must occur in the context of an ensemble of neurons (Mayford et al., 2012). Hebb theorized that cells repeatedly active simultaneously would become associated with each other calling them cell assemblies (Hebb, 1949). Notably, the fate of a memory trace is not necessarily determined at the time of encoding (Nomoto et al., 2016) but is instead influenced by synaptic plasticity mechanisms present to modulate that trace. The strength of a memory trace, which can act as a *boundary condition* for reconsolidation of a memory to determine its susceptibility to modification, is directly correlated with the neuromodulatory actions of the LC-NE system at encoding (Haubrich et al., 2020). This is also evident in models of *synaptic tag and capture* or *behavioral tagging* (Redondo and Morris, 2011; Nomoto et al., 2016) wherein less prominent events are more effectively remembered simply due to their proximity in time to a significant event. When memories are linked there is a higher degree of overlap in the ensembles that make up their respective engrams (Rogerson et al., 2014; Grella et al., 2022; Zaki et al., 2022). Blocking D1/D5 receptors has been shown to suppress this overlap and impair synaptic tagging (Nomoto et al., 2016). Additionally, Chowdhury et al. (2022) found that dopaminergic inputs from the LC to the dorsal CA1 constituted an important pathway in memory linking to sustain ensemble overlap through the maintenance of cellular excitability and ensemble firing. These effects were independent of hippocampal adrenergic activity suggesting that LC-NE inputs may mediate novelty and novelty enhancing effects to induce encoding while LC-DA inputs may gate salience and play a role in memory linking. However, they also found that inhibiting DA inputs from the LC to the dorsal CA3 did not affect memory linking but did affect memory formation. Interestingly, Wagatsuma et al. (2018) found that in a test for novel context recognition, infusions of SCH23390 but not propranolol into the CA3, were able to impair contextual learning. They also found that inhibiting the LC did not affect the size of the contextual engrams formed within the DG and the CA3 but did reduce this size in the CA1. Given that the CA3 projects to the CA1, this is consistent with an impairment in encoding in the CA3 for novel contexts. Additionally, silencing the LC resulted in a reduced capacity for the CA3 and the CA1 to reactivate the original engram cells upon re-exposure to the context signaling a deficit in ensemble dynamics. A comprehensive understanding requires further research, but there is a possibility that LC inputs to the DG/CA3 regions play a more significant role in encoding than LC-CA1 projections. Additionally, the processing of novelty might be influenced more by NE in LC-DG projections and by DA in LC-CA3 projections. Nevertheless, both LC NE and DA neuromodulatory systems appear to work in concert to promote memory updating and cognitive and behavioral flexibility in an adaptive manner. For instance, synaptic tagging involving LTP has been shown to require both D1/D5 and βARs (Sajikumar et al., 2005; O’Carroll et al., 2006).

### 4.5 Hippocampal catecholamine release and memory consolidation

The majority of studies that have examined the role of NE on memory have looked at post-encoding effects, namely on consolidation and reconsolidation, specifically in the context of emotional modulation of memory (van Stegeren et al., 1998; Przybyslawski et al., 1999; Cahill and Alkire, 2003). While most of these studies found that NE enhances consolidation and reconsolidation, these effects were mostly attributed to activation of βARs in the BLA (Ferry et al., 1999; McGaugh, 2000; Roozendaal et al., 2009; Roozendaal and McGaugh, 2011; Gazarini et al., 2013). Moreover, administration of propranolol has produced inconsistent results over the years in both animals and humans and the memory-impairing effects that have been observed are likely due to alterations in the emotional valence of the memory rather than a disruption of the contextual elements of the memory trace (Villain et al., 2016).

One of the main functions of NE is to increase responsiveness to novel stimuli (Berridge and Waterhouse, 2003; Benarroch, 2009; Sara, 2009), and this has an enhancing effect on memory consolidation (Roozendaal and Hermans, 2017) and may in fact involve an interaction between NE and DA. Takeuchi et al. (2016) found that intermittent burst stimulation of the LC using optogenetics given 30 min post-encoding, resulted in enhanced consolidation of a spatial memory. When they applied post-encoding intrahippocampal micro-infusions of propranolol vs. SCH23390, they found that only SCH23390 blocked this enhancement. The authors introduced the idea that either LC terminals co-release NE and DA in the hippocampus or these effects may involve heterodimerization of NE and DA receptors (Perreault et al., 2014). Different types of memory may be modulated differently by NE and DA. Bevilaqua et al. (1997), investigated the impact of hippocampal CA1 and amygdalar pharmacological manipulations on inhibitory avoidance learning. NE infusion into the CA1 enhanced memory when given immediately post-learning, diminishing at 1.5 h but persisting at 3 or 6 h. However, the effect was lost at 9 h. SCH23390 administered 3- or 6-h post-learning induced retrograde amnesia. Notably, within the amygdala, NE facilitated memory only when administered immediately after learning. The authors pointed to a hippocampal cAMP/protein kinase A pathway crucial to memory consolidation at 3 and 6 h from training regulated by βARs and D1 receptors. This study, among others, underscores the temporal nuances of pharmacological interventions in specific brain regions, highlighting the critical role of timing in memory processes during learning, suggesting that the effect of modulating catecholamines on memory will critically depend on the stage of training (Grella et al., 2021).

In a study involving electrolytic lesions of the LC in mice, mice were tested on a one-trial inhibitory avoidance step-through task (Zornetzer and Gold, 1976). Mice received lesions immediately after training and were tested for retention 48 h later with no impairments. Using the same procedure, a separate set of mice, received transcorneal electroconvulsive shock either 40 h after, or 7 days after training and lesions were tested for retention either 8 or 24 h later. They found that only the 40-h group showed disrupted performance. These results imply that the LC plays a crucial role in memory consolidation. When the LC is offline or not actively engaged, memories appear to be more vulnerable to interference, and factors contributing to amnesia may exert a more pronounced influence.

Memory consolidation involves post-learning, off-line reactivation of memory traces during slow wave sleep (SWS) (Buzsáki, 1989; Wilson and McNaughton, 1994). The concept of memory traces being supported by asynchronous reactivation was first postulated by Marr (1971). During these off-line states, sharp wave ripple (SWR) complexes are observed in the hippocampus. Particularly when lasting over 100 milliseconds, they indicate engagement in a novel environment or memory-related task. They are characterized by increased cellular activity (up states) (Buzsáki, 1985) and the replaying of neuronal sequences that were active during a previous experience (Kudrimoti et al., 1999). Interestingly, although LC neurons are typically not active during sleep, they do exhibit intermittent bursts of discharge during SWS following learning Eschenko and Sara (2008) and Eschenko et al. (2012) found that this discharge precedes upstate. Thus, the LC may exert modulatory influence over memory consolidation during sleep. Within the hippocampus, βAR activation has been shown to enhance SWRs facilitating memory consolidation while α1 adrenoreceptor activation has the opposite effect (Ul Haq et al., 2016). D1/D5 receptor activation, is also implicated in SWR facilitation, leading to an augmentation of sharp wave events (Miyawaki et al., 2014). While these findings suggest that the neuromodulation of both NE and DA influences SWRs, more research is needed to fully understand these relationships.

## 5 Concluding perspectives and future research directions

Fifty years ago, Kety (1970) proposed that catecholamines acts as neuromodulators, particularly in enhancing memory during emotionally significant events. Numerous studies since then have demonstrated the role of NE and DA in facilitating the consolidation and retention of emotional memories over hours to days, dependent on βARs. Peri-encoding activation and inactivation of the LC, the major source of both NE and DA in the hippocampus, promotes plasticity of hippocampal-dependent memory via changes in ensemble dynamics involving the transcription of plasticity related genes and remapping of contextual representations. The findings presented here also support a particular role for LC NE/DA projections to the hippocampus in modulating memory on shorter time scales, as well as assigning new networks to mediate encoding that reflects environmental change and may constitute an important pathway involved in memory updating. Consistent with Kety (1970) original hypothesis, we assert that NE and DA, through LC activation during significant events, are instrumental in acquiring new information and encoding memories to form new memories or update existing ones. Phasic LC NE/DA discharge is associated with disengagement of established representations, enhancing processes that favor the incorporation of new information and encoding novel hippocampal sequences. Following the network reset hypothesis (Bouret and Sara, 2005), we extend this to include a mnemonic bias toward encoding rather than retrieval during adaptive conditions that often require cognitive shifts, promoting both cognitive and behavioral flexibility. Detecting and responding adaptively to salient stimuli is crucial for survival, particularly in uncertain circumstances. The LC plays a role in initiating cognitive shifts in attention (Sara, 2009; Rorabaugh et al., 2017). Studies on LC target projections in Attention Deficit/Hyperactivity Disorder (ADHD) are growing, suggesting potential avenues for intervention (Brennan and Arnsten, 2008; Arnsten and Pliszka, 2011; Berridge and Devilbiss, 2011; Darcq and Kieffer, 2015).

While a focus of this paper has been on the neuromodulatory influence of LC activity on hippocampal engrams, it is worth considering that theories of consolidation such as *systems consolidation* (Squire and Alvarez, 1995; Squire et al., 2015) involving the reorganization of memory traces to support long term remote memory highlight the role of contextual engrams within cortical areas such as the PFC. Moreover, LC projections to other regions such as the BLA, the periaqueductal gray (PAG) and the insula may also be important for contextual memory and engram stability and flexibility. The LC densely innervates these areas as well as the hippocampus (Arnsten and Goldman-Rakic, 1984). For instance, it has been shown that noradrenergic signaling during CFC is important for the recruitment of engrams in the PFC early on and remains important for remote memory processes. Blocking NE neurotransmission impairs this ability and is not easily rescued with LC activation. Moreover, the expression of freezing representing a CFC memory was regulated by the LC-PAG pathway (Fan et al., 2022). It will be interesting to see how future studies explore these other coerulear projections in terms of engram dynamics as well as examine the similarities and differences between LC neuromodulatory actions in these regions as compared to the hippocampus. Examining the interaction between these circuits will be crucial to a better understanding of how the LC supports and modulates contextual memory.

It is important to note, that overactivation of the LC such as in chronic or traumatic stress, can reduce plasticity in part by strengthening stress-related memories, which then affects boundary conditions of memory reconsolidation processes such that these memories are more resistant to modification. Consequently, understanding LC dynamics is vital for addressing anxiety disorders such as PTSD. The LC plays a key role in conditioned fear, a process tied to fear-related contextual memory in the hippocampus (Davis, 1986; Bremner et al., 1996). In PTSD, the recall of traumatic events, dominated by fear-related memories, may involve NE release in the hippocampus (Bremner et al., 1996). Individuals with PTSD may struggle with cognitive shifts, reactivating fear-related representations in non-fear related contexts, hindering adaptive behavior (Strawn and Geracioti, 2008; Sara, 2016). Multiple recent studies, including our own yet-to-be-published findings, have endeavored to manipulate hippocampal-dependent contextual memory by activating LC-hippocampus pathways to promote cognitive and behavioral flexibility that might be compromised in certain models associated with severe/chronic stress or repeated drug use during periods of new learning. For instance, the LC is implicated during early extinction, and optical activation of LC to DG projection neurons just before administering a recall-extinction procedure in morphine-dependent mice, resulted in enhanced extinction (Dai et al., 2023). We are also currently exploring the role of LC activation on fear memory reconsolidation (Asgarali et al., 2023) and the enhancement of extinction learning. It is logical that the LC would be involved during early extinction since this phase poses the highest likelihood for a prediction error to occur.

Likewise, the LC also plays a role in reversal learning (Sara, 2009). One study in particular (Rorabaugh et al., 2017), was able to rescue deficits in reversal learning in TgF344-AD rats, where hyperphosphorylated tau was detected in the LC prior to accrual in the mEC or hippocampus, via chemogenetic activation of the LC. Hallmark characteristics of Alzheimer’s disease (AD) include the presence of amyloid beta plaques, the formation of insoluble aggregates referred to as neurofibrillary tangles (NFTs) arising from the hyperphosphorylation of tau protein (Huber et al., 2023), neuroinflammation, and neuronal cell death (Selkoe, 1997). Braak staging is a topographic representation of the development of NFTs and therefore AD progression (Braak and Braak, 1991). Until recently, AD was predominantly considered a cortical pathology characterized by retrograde tau pathology spread to subcortical areas. However, emerging research reveals those subcortical structures, particularly the serotonergic dorsal raphe nucleus and noradrenergic/dopaminergic LC, exhibit AD-type tau aggregates even preceding the EC which occur in Braak stage II (Vogels et al., 2020). Given the LC’s extensive projections to the hippocampus, it is unspringing that it has widespread involvement in AD-related memory impairment. The presence of these aggregates has detrimental effects and correlates with cognitive decline. These changes are not ascribed to normal aging. While research on aging and cognitive decline have traditionally focused on the impact of cortical pathology, there has been a shift to study the crucial modulatory roles played by these subcortical structures. Given the proximity of the LC to the fourth ventricle, the LC is a region highly sensitive to toxins and infections via exposure to cerebral spinal fluid, and has been shown to exhibit AD-related pathology very early on in the progression of the disease (Mather and Harley, 2016). Therefore, LC integrity, specifically the rostral LC, may capture early aberrant signs of AD and may predict clinical symptomology (Van Egroo et al., 2023) even though the LC does not undergo significant neuronal loss before Braak stage IV (Theofilas et al., 2017). Additionally, the LC is implicated in neuroinflammation, a process heightened in AD (Kinney et al., 2018), and plays a role in amyloid-beta clearance (Ross et al., 2015). Dysfunction of the LC may contribute to impaired clearance mechanisms, leading to amyloid-beta accumulation and plaque formation (Ross et al., 2015; Mather and Harley, 2016). The integrity of the LC is also associated with cognitive reserve, influencing the brain’s ability to maintain function despite pathology (Mather and Harley, 2016). Ongoing research seeks to elucidate the complex mechanisms linking LC dysfunction to various aspects of AD pathology and cognitive decline. Recent studies, such as the one conducted by Rorabaugh et al. (2017), provide optimism regarding the prospect of utilizing LC activation studies to gain insights into the involvement of this structure in the aging brain. This approach holds the potential for more precise and targeted treatments as well as improved detection methods for AD and other neurodegenerative disorders.

Thus, navigating the complex landscape of neuroscience, the LC emerges as a central player in the intricate puzzle of both AD and PTSD. In AD, where memories deteriorate and neuronal atrophy is evident, the challenge lies in decoding how disruptions in the LC, possibly linked to tau pathology, contribute to cognitive decline. Simultaneously, in PTSD, trauma-related memories persist unwelcomingly, often reactivating spontaneously in inappropriate contexts to produce fear generalization. However, beyond the realm of memory dysfunction, both disorders converge on another shared feature—the loss of cognitive flexibility. Building upon Kety (1970) original hypothesis and the subsequent investigations by scientists delving into the function of the LC on cognition, the LC has gained prominence for regulating cognitive processes. It stands as a focal point for understanding cognitive impairment. Exploring the nuanced connections between LC dysfunction, compromised cognitive flexibility, and the LC’s influence on memory formation and maintenance in both disorders offers a comprehensive perspective. This underscores the imperative for a unified theory to unravel the intricate interplay of the LC in shaping cognitive processes across diverse neurological contexts. This pursuit could provide crucial insights into the mechanisms underlying both diseases and opens avenues for targeted therapeutic interventions in the realm of neurodegenerative and trauma-related disorders.

## Author contributions

SG: Conceptualization, Data curation, Funding acquisition, Investigation, Project administration, Supervision, Writing – original draft, Writing – review and editing. TD: Data curation, Investigation, Writing – original draft.
